# CoBaltDB: Complete bacterial and archaeal orfeomes subcellular localization database and associated resources

**DOI:** 10.1186/1471-2180-10-88

**Published:** 2010-03-23

**Authors:** David Goudenège, Stéphane Avner, Céline Lucchetti-Miganeh, Frédérique Barloy-Hubler

**Affiliations:** 1 CNRS UMR 6026, ICM, Equipe B@SIC, Université de Rennes 1, Campus de Beaulieu, 35042 Rennes, France

## Abstract

**Background:**

The functions of proteins are strongly related to their localization in cell compartments (for example the cytoplasm or membranes) but the experimental determination of the sub-cellular localization of proteomes is laborious and expensive. A fast and low-cost alternative approach is *in silico *prediction, based on features of the protein primary sequences. However, biologists are confronted with a very large number of computational tools that use different methods that address various localization features with diverse specificities and sensitivities. As a result, exploiting these computer resources to predict protein localization accurately involves querying all tools and comparing every prediction output; this is a painstaking task. Therefore, we developed a comprehensive database, called CoBaltDB, that gathers all prediction outputs concerning complete prokaryotic proteomes.

**Description:**

The current version of CoBaltDB integrates the results of 43 localization predictors for 784 complete bacterial and archaeal proteomes (***2.548.292 proteins in total***). CoBaltDB supplies a simple user-friendly interface for retrieving and exploring relevant information about predicted features (such as signal peptide cleavage sites and transmembrane segments). Data are organized into three work-sets ("*specialized tools*", "*meta-tools*" and "*additional tools*"). The database can be queried using the organism name, a locus tag or a list of locus tags and may be browsed using numerous graphical and text displays.

**Conclusions:**

With its new functionalities, CoBaltDB is a novel powerful platform that provides easy access to the results of multiple localization tools and support for predicting prokaryotic protein localizations with higher confidence than previously possible. CoBaltDB is available at http://www.umr6026.univ-rennes1.fr/english/home/research/basic/software/cobalten.

## Background

Determining the subcellular localization of proteins is essential for the functional annotation of proteomes [1,2]. Bacterial proteins can exist in soluble (*i.e *free) forms in cellular spaces (cytoplasm in both monoderm and diderm bacteria and periplasm in diderms), anchored to membranes (cytoplasm membrane in monoderms, inner- or outer membrane in diderms) or cell wall (in monoderms). They can also be released into the extracellular environment or directly translocated into host cells [3]. All protein synthesis takes place in the cytoplasm, so all non-cytoplasmic proteins must pass through one or two lipid bilayers by a mechanism commonly called "secretion". Protein secretion is involved in various processes including plant-microbe interactions [4,5]), biofilm formation [6,7] and virulence of plant and human pathogens [8-10]. Two main systems are involved in protein translocation across the cytoplasmic membrane, namely the essential and universal Sec (Secretion) pathway and the Tat (Twin-arginine translocation) pathway found in some prokaryotes (monoderms and diderms) and eukaryotes alike [11-16]. The Sec machinery recognizes an N-terminal hydrophobic signal sequence and translocates unfolded proteins [12], whereas the Tat machinery recognizes a basic-rich N-terminal motif (SRR-x-FLK) and transports fully folded proteins [13,14]). In addition to these systems, diderm bacteria have six further systems that secrete proteins using a contiguous channel spanning the two membranes (T1SS, [17,18], T3SS, T4SS and T6SS [19-24]) or in two steps, the first being Sec- or Tat-dependent export into the periplasmic and the second being translocation across the outer membrane (T2SS, [25-27] and T5SS, [28,29]). Other diderm protein secretion systems exist: they include the chaperone-usher system (CU or T7SS, [30,31]) and the extracellular nucleation-precipitation mechanism (ENP or T8SS, [32]). It is worth mentioning that the terminology T7SS has also been proposed to describe a completely different protein secretion system, namely the ESAT-6 protein secretion (ESX) in Mycobacteria, now considered as diderm bacteria [33]. Beside Sec and Tat pathways, monoderm bacteria have additional secretion systems for protein translocation across the cytoplasmic membrane, namely the flagella export apparatus (FEA [34]), the fimbrilin-protein exporter (FPE, [35,36]) and the WXG100 secretion system (Wss, [37,38]).

Establishing whole proteome subcellular localization by biochemical experiments is possible but arduous, time consuming and expensive. Data concerning predicted proteins (from whole genome sequences) is continuously increasing. High-throughput *in silico *analysis is required for fast and accurate prediction of additional attributes based solely on their amino acid sequences. There are large numbers of global (that yield final localization) and specialized (that predict features) tools for computer-assisted prediction of protein localizations. Most specialized tools tend to detect the presence of N-terminal signal peptides (SP). Prediction of Sec-sorting signals has a long history as the first methods, based on weight matrices, were published about fifteen years ago [39-41]. Numerous machine learning-based methods are now available [42-50]. The distinction between Tat- and Sec- sorting signals is essentially based on the recognition, in the n/h regions edge, of the twin-arginine motif [51], using regular expressions combined with hydrophobicity measures [52] or machine learning [53]. Pre-lipoproteins SP have the same n- and h- regions as Sec SP but contain, in the c-region, a well-conserved lipobox [54], recognized for cleavage by the type II signal peptidase [55]. Lipoprotein prediction tools use regular expression patterns to detect this lipobox [56,57], combined with Hidden Markov Models (HMM) [58] or Neural Networks (NN) [59]. Other attributes predicted by specialized tools are α-helices and β-barrel transmembrane segments. In 1982, Kyte and Doolittle proposed a hydropathy-based method to predict transmembrane (TM) helices in a protein sequence. This approach was enhanced by combining discriminant analysis [60], hydrophobicity scales [61-63] amino acid properties [64,65]. Complex algorithms are also available and employ statistics [66], multiple sequence alignments [67] and machine learning approaches [68-73]. β-barrel segments, embedded in outer membrane proteins, are harder to predict than α-helical segments, mostly because they are shorter; nevertheless, many methods are available based on similar strategies [74-87].

This plethora of protein localization predictors and databases [88-91] constitutes an important resource but requires time and expertise for efficient exploitation. Some of the tools require computing skills, as they have to be locally installed; others are difficult to use (numerous parameters) or to interpret (large quantities of graphics and output data). Web tools are disseminated and need numerous manual requests. Additionally, researchers have to decide which of these numerous tools are the most pertinent for their purposes, and selection is problematic without appropriate training sets. Recent work shows that the best strategy for exploiting the various tools is to compare them [92-94].

Here, we describe CoBaltDB, the first public database that displays the results obtained by 43 localization predictor tools for 776 complete prokaryotic proteomes. CoBaltDB will help microbiologists explore and analyze subcellular localization predictions for all proteins predicted from a complete genome; it should thereby facilitate and enhance the understanding of protein function.

## Construction and content

### Data sources

The major challenge for CoBaltDB is to collect and integrate into a centralized open-access reference database, non-redundant subcellular prediction features for complete prokaryotic orfeomes. Our initial dataset contained 784 complete genomes (731 bacteria and 53 Archaea), downloaded with all plasmids and chromosomes (1468 replicons in total), from the NCBI ftp server ftp://ftp.ncbi.nih.gov/genomes/Bacteria in mid-December 2008. This dataset contains 2,548,292 predicted non-redundant proteins (Additional file 1).

The CoBaltDB database was designed to associate results from disconnected resources. It contains three main types of data: *i*) CoBaltDB pre-computed prediction using 23 feature-based localization tools (Table 1), *ii*) CoBaltDB pre-computed prediction obtained using 5 localization meta-tools (Table 2) and *iii*) data collected from 20 public databases with both predicted and experimentally determined subcellular protein localizations (Table 3).

**Table 1:** A summary of CoBaltDB precomputed features-tools

Program	Reference	Analytical method	CoBaltDB features prediction group(s)				
LipoP 1.0 Server	[59]	HMM + NN	LIPO		SEC		
DOLOP	[57]	RE	LIPO				
LIPO	[56]	RE	LIPO				
TatP 1.0	[53]	RE + NN		TAT			
TATFIND 1.4	[52]	RE		TAT			
PrediSi	[112]	Position weight matrix			SEC		
SignalP 3.0 Server	[45-47]	HMM + NN			SEC		
SOSUIsignal	[113]	Multi-programs			SEC		
SIG-Pred	J.R. Bradford	Matrix			SEC		
RPSP	[44]	NN			SEC		
Phobius	[48,49]	HMM			SEC	αTMB	
HMMTOP	[71]	HMM				αTMB	
TMHMM Server v.2.0	[70]	HMM				αTMB	
TM-Finder	[65]	AA FEATURES				αTMB	
SOSUI	[114]	AA FEATURES				αTMB	
SVMtm	[73]	SVM				αTMB	
SPLIT 4.0 Server	[115]	AA FEATURES				αTMB	
MCMBB	[116]	HMM					βBarrel
TMBETADISC:	[117]						
_COMP		AA FEATURES					βBarrel
_DIPEPTIDE		Dipeptide composition					βBarrel
_MOTIF		Motif(s)					βBarrel
TMB-Hunt2	[118]	SVM					βBarrel

HMM: Hidden Markov Model, NN: Neural Network, RE: Regular Expression, AA: Amino Acid, SVM: Support Vector Machine

**Table 2:** A summary of CoBaltDB precomputed meta-tools

Program	Reference	Analytical method	Localizations
Subcell Specialization Server 2.5	[119]	Multiple classifiers	5 diderms/3 monoderms
SLP-Local	[120]	SVM	3 with no distinction
SubLoc v1.0	[121]	SVM	3 with no distinction
Subcell (Adaboost method)	[122]	AdaBoost algorithm	3 with no distinction
SOSUIGramN	[123]	Physico-chemical parameters	5 diderms/no monoderm

SVM: Support Vector Machine

**Table 3:** A summary of CoBaltDB integrated databases and tools features.

Databases	Reference	Features predicted	Genome(s)	Protein numbers
EchoLOCATION	[124]	Subcellular-location (EXP)	*E. coli K-12*	4330 (506 exp)
Ecce	_	Subcellular-location	*E. coli K-12*	306
LocateP DataBase	[89]	Subcellular-location	178 MD	542788
cPSORTdb	[91]	Subcellular-location	140 BA	1634278
ePSORTdb	[91]	Subcellular-location (EXP)		2165
THGS	[125]	Transmembrane Helices	689 PROK	465411
Augur	[88]	Subcellular-location	126 MD	111223
CW-PRED	[126]	Cell-anchored (surface)	94 MD	954
PROFtmb	[78]	Beta-barrel (OM)	78 DD/19 MD	2152
HHomp	[127]	Beta-barrel (OM)		12495
PRED-LIPO	[58]	Lipoprotein SPs	179 MD	895
SPdb	[90]	Signal peptides (SPs)	855 PROK	7062
ExProt	[128]	Signal peptides (SPs)	23 AR/61 MD/115DD	
Signal Peptide Website	_	Signal peptides (SPs)	384 BA	1161 (EXP)
PRED-SIGNAL	[129]	Signal peptides (SPs)	48 AR	9437
TMPDB	[130]	Alpha Helices & Beta-barrel		188
DTTSS	Shandong Univ.	Type III secretion system		1035
TOPDB	[131]	Transmembrane Proteins	755 BA/16 AR	
TMBC-Database	Andrew Garrow	Transmembrane Beta-barrel		1219
Swissprot signal testset	[132]	Signal peptides (SPs) (EXP)	176 SP+/122 SP-	

MD = monoderm, DD = diderm. AR (Archeae), BA (Bacteria), PROK (Prokaryotes) include both bacteria and Archaee, EXP = Experimental database

These data were organized in five "*boxes*" with regard to the features predicted: three boxes correspond to signal peptide detection (Lipoprotein, Tat- and Sec- dependent targeting signals); one box for the prediction of alpha-transmembrane segments (TM-Box); and one box, only available for diderms (Gram-negatives), for outer membrane localization through prediction of beta-barrels.

### Data generation

There is a great diversity of web and stand-alone resources for the prediction of protein subcellular location. We retrieved and tested 99 currently (in 2009) available specialized and global tools (software resources) that use various amino acid features and diverse methods: algorithms, HMM, NN, Support Vector Machine (SVM), software suites and others), to predict protein subcellular localization (Additional file 2). All tools were evaluated: some are included in CoBaltDB, some may be launched directly from the platform (Table 4), and others were excluded because of redundancy or processing reasons or both (Table 5). Some tools are specific to Gram-negative or Gram-positive bacteria. Many prediction methods applicable to both Gram categories have different parameters for the two groups of bacteria. For these reasons, each NCBI complete bacterial and archaeal genome implemented in CoBaltDB was registered as "monoderm" or "diderm", on the basis of information in the literature and phylogeny (Additional file 3). Monoderms and diderms were considered as Gram-negative and Gram-positive, respectively. All archaea were classified as monoderm prokaryotes since their cells are bounded by a single cell membrane and possess a cell envelope [3,95]. An exception was made for *Ignicoccus hospitalis *as it owns an outer sheath resembling the outer membrane of gram-negative bacteria [96].

**Table 4:** Tools available using CoBaltDB "post" window

Program	Reference	Analytical method	CoBaltDB features prediction group(s)			
LipPred	[133]	Naive Bayesian Network	LIPO			
PRED-LIPO	[58]	HMM	LIPO		(only Monoderm)	
SPEPLip	[134]	NN	LIPO	SEC		
SecretomeP	[135]	Pattern & NN		ΔSEC_SP		
Signal-3L	[136]	Multi-modules		SEC		
Signal-CF	[137]	Multi-modules		SEC		
Signal-Blast	[138]	BlastP		SEC		
Sigcleave	EMBOSS	Von Heijne method		SEC		
PRED-SIGNAL	[129]	HMM		SEC	(only Archae)	
Flafind	[139]	AA features		T3SS Archae + T4SS Bacteria		
T3SS_prediction	[110]	SVM & NN		T3SS		
EffectiveT3	[111]	Machine learning		T3SS		
NtraC Signal Analysis	[140]	Pattern model		SEC (long SP)		
Philius	[141]	HMM		SEC	αTMB	
(SP)OCTOPUS	[142,143]	Blast Homology, NN, HMM		SEC	αTMB	
MemBrain	[144]	Machine learning		SEC	αTMB	
DAS	[145]	Dense Alignment Surface			αTMB	
HMM-TM	[146]	HMM			αTMB	
SVMtop Server 1.0	[147]	SVM			αTMB	
UMDHMM_TMHP	[148]	HMM			αTMB	
waveTM	[149]	Hydropathy signals algorithm			αTMB	
PRED-TMR	[150]	AA features			αTMB	
TMAP	[67]	AA features			αTMB	
igTM	[151]	Grammatical Inference			αTMB	
TOPCONS	[152]	Tools Consensus			αTMB	
TUPS	[153]	Tools Consensus			αTMB	
ConPred II	[154]	Tools Consensus			αTMB	
MEMSAT3	[66,155]	NN			αTMB	
SABLE	[156]	NN			αTMB	
TM-Pro	[64,157]	AA features			αTMB	
ProspRef	_	Knowledge-based method			αTMB	
PSIPRED	[158,159]	NN, PSSM			αTMB	
NPS@	[160]	Tools Consensus			αTMB	
SAM-T08	[161]	HMM			αTMB	
PORTER	[162]	NN			αTMB	
TMPred	EMBnet	Weight-matrices			αTMB	
TMMOD	[163]	HMM			αTMB	
TopPred II	[61]	G. von Heijne algorithm			αTMB	
YASPIN	[164]	Hidden Neural Network			αTMB	
MemType-2L	[165]	PseudoPSSM, classifier			Membrane Type	
BOMP	[84]	AA features				βBarrel
TMBETADISC-RBF	[87]	RBF network, PSSM				βBarrel
TMBETA-NET	[117]	AA features				βBarrel
PRED-TMBB	[85]	HMM				βBarrel
ConBBPred	[76]	Tools Consensus				βBarrel
CW-PRED (submit)	[126]	HMM		Cell-Wall (only Monoderm)		
ProtCompB	SoftBerry	Multi-methods	Localization			
CELLO	[166]	SVM	Localization			
PSL101	[167]	SVM, structure homology	Localization			
PSLpred	[168]	SVM	Localization			
GPLoc-neg	[169]	Basic classifier	Localization		(only Diderm)	
GPLoc-pos	[170]	Basic classifier	Localization		(only Monoderm)	
LOCtree	[171]	SVM	Localization			
PSORTb	[91]	Multi-modules	Localization			
SLPS	[172]	Nearest Neighbor on domain	Localization			
Couple-subloc v1.0	Jian Guo	AA features	Localization			
TBPRED	[173]	SVM	Localization		(only *Mycobacterium*)	

HMM: Hidden Markov Model, NN: Neural Network, AA: Amino Acid, SVM: Support Vector Machine, PSSM: Position Specific Scoring Matrix, T3SS: Type III Secretion System, RBF: Radial Basis Function

**Table 5:** Tools and Database not available in CoBaltDB

Program	Reference	Analytical method	CoBaltDB features prediction group(s)			
SpLip	[174]	Weight matrix	LIPO		(only *Spirochaetal*)	
PROTEUS2	[175]	Multi-Methods		SEC	αTMB	βBarrel
PRED-TMR2	[176]	NN			αTMB	
PRODIV-TMHMM	[72]	Multi HMM			αTMB	
S_TMHMM	[72]	HMM			αTMB	
TransMem	[69]	NN			αTMB	
BPROMPT	[177]	Bayesian Belief Network			αTMB	
orienTM	[178]	Statistical analysis			αTMB	
APSSP2	[179]	Multi-Methods			Secondary structure	
PRALINE_TM	[180]	Alignment, tools consensus			Secondary structure	
OPM (DB)	[181]	Multi-Methods			Membrane orientation	
MP_Topo (DB)	[182]	Experimental			TMB	
PDBTM (DB)	[183]	TMDET algorithm			TMB	
TMB-HMM	A.Garrow	HMM, SVM				βBarrel
TMBETA-SVM	[86]	SVM				βBarrel
TMBETA-GENOME (DB)	[184]	Multi-Methods				βBarrel
PredictProtein	[185]	Alignment, Multi-Methods	Localization			
EcoProDB (DB)	[186]	Identification on 2D gels	Localization		(only *E.coli*)	
LOCTARGET (DB)	[187]	Multi-Methods	Localization			
DBMLoc (DB)	[188]	_	Localization			

NN: Neural Network, HMM: Hidden Markov Model, SVM: Support Vector Machine

**Figure 1:**
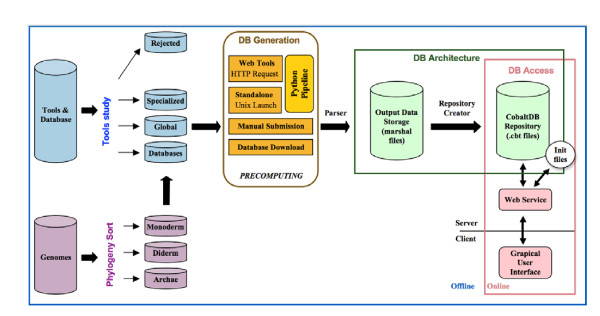
**A schematic view of the CoBaltDB workflow**. CoBaltDB integrates the results of 43 localization predictors for 784 complete bacterial and archaeal proteomes. Each complete NCBI prokaryotic genome implemented in CoBaltDB was classified as: archaea, or monoderm or diderm bacteria. 101 protein subcellular location predictors were evaluated and few were rejected. Selected tools were classified as: feature localization tools (Specialized), localization meta-tools (Global) or databases. The data recovery process was performed manually or via a Web automat using a python automatic submission workflow for both stand-alone and web-based tools. Databases were downloaded. For each protein, ouptuts collected were parsed and selected items were stored in particular CoBaltDB formatted files (.cbt). The parsing pipeline creates one ".cbt" file per replicon to compose the final CoBaltDB repository. The client CoBaltDB Graphical User Interface communicates with the server-side repository via web services to provide graphical and tabular representations of the results.

Currently, CoBaltDB contains pre-computed results obtained with 48 tools and databases, and additionally provides pre-filled access to 50 publicly available tools that could not be pre-computed or that provide new information (tools dedicated to a special phylum, consensus tools or tools predicting proteins secreted via other pathways). The data pre-computing process is illustrated in Figure 1; web-based and stand-alone tools were used separately. Web-based localization prediction tools were requested via a Web automat, a python automatic submission workflow using both "httplib" and "urllib" libraries. A different script was created for each tool. For web-tools with no equivalent (such as "TatP" for Tat-BOX and "LIPO" for Lipoprotein-BOX) and incompatible with automatic requests, we collected results manually. CoBaltDB also provides a platform with automatically pre-filled forms for additional submissions to a selection of fifty recent or specific web tools (Table 4). The stand-alone tools were installed on a Unix platform (unique common compatible platform) and included in a global python pipeline with the HTTP request scripts. We selected information from a up-to-date collection of 20 databases and integrated this data within CoBaltDB; these databases were retrieved by simple downloading or creating an appropriate script which navigates on the web databases to collect all protein information. The global python pipeline used multi-threading to speed up the pre-computation of the 784 proteomes.

### Database Creation and Architecture

For each protein, every output collected (a HTML page for web tools and a text file for standalone applications) was parsed and selected items were stored in a particular format: binary "marshal" files. The object structure obtained by parsing tool output was directly saved into a marshal file, allowing a quick and easy opening by directly restoring the initial parsing object. Another script then creates the CoBaltDB repository, by reading and analysing all marshal files to generate a specific formatted file (".cbt") for each replicon. These files contain all the required protein information and a simplified representation of the tools' results. Some initialization files containing information about phylogeny or genome features are also used.

The repository is used by the Graphical User Interface (GUI) to display CoBaltDB information. For raw data from tools, the GUI accesses the marshal file directory.

### Accessing the CoBaltDB Repository and Raw Data

The CoBaltDB platform has been developed as a client-server application. The server is installed at the Genouest Bioinformatics platform http://www.genouest.org/?lang=en. The client is a Java application that needs to be locally downloaded by the users. Queries are submitted to the server-side CoBaltDB repository using a locally installed client GUI that provides tabular and graphical representations of the data. The repository is accessed through SOAP-based web services (Simple Object Access Protocol), implemented in Java 5 using the Apache Axis 1.4 toolkit and deployed on the servlet engine Tomcat 5.5.20. CoBaltDB integrates: an initialization web service (that returns the current list of genomes supported); two repository web services that allow querying the database either by specifying a replicon or a list of locus tags; and a raw data web service that retrieves all recorded raw data generated by a given tool for the specified locus tag.

## Utility

### Running CoBaltDB

Our goal was to build an open-access reference database providing access to protein localization predictions. CoBaltDB was designed to centralize different types of data and to interface them so as to help researchers rapidly analyse and develop hypotheses concerning the subcellular distribution of particular protein(s) or a given proteome. This data management allows comparative evaluation of the output of each tool and database and thus straightforward identification of inaccurate or conflicting predictions.

**Figure 2:**
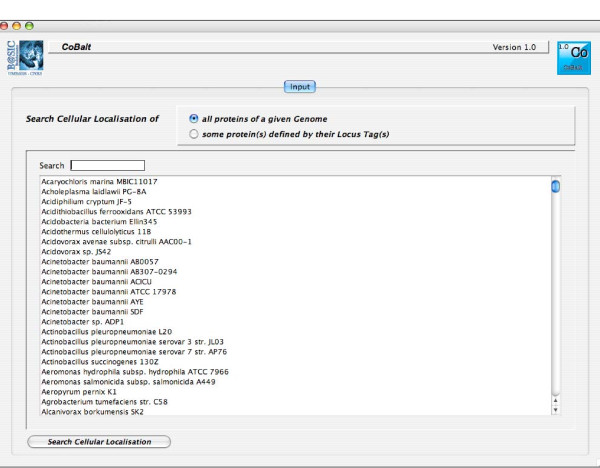
**A snapshot of the CoBaltDB input interface**. The "input" module allows the selection of organisms, using organism name completion or through an alphabetical list. Users can also enter a subset of proteins, specified by their locus tags.

**Figure 3:**
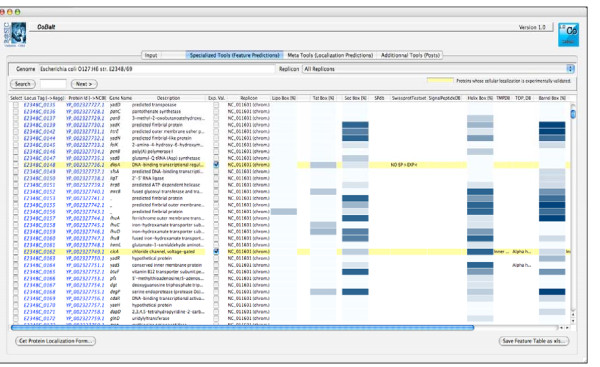
**The CoBaltDB Specialized Tools viewer**. The "Specialized tools" browser supplies a tabular output for every protein, enriched with the protein's annotation including locus tag, protein identifier, gene name (if available) and product descriptions. Clicking on each "locus tag" opens a navigator window with related KEGG link whereas clicking on every "protein Id" opens the corresponding NCBI entry web page. Clicking on the white/blue heat map reveals the raw results of all tools corresponding to the feature box considered.

**Figure 4:**
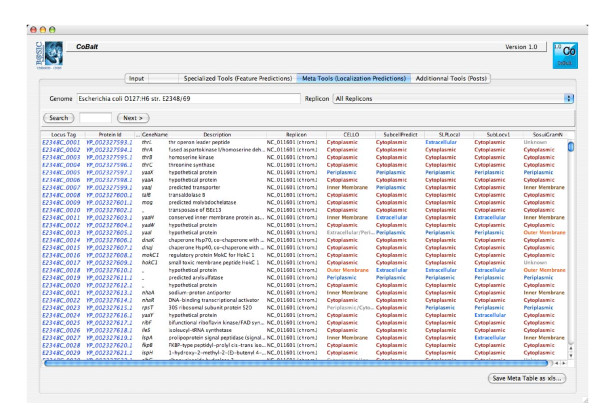
**The CoBaltDB Meta-Tools interface**. The "meta-tools" panel presents the CoBaltDB-computed results for multi-modular prediction software that uses various techniques to directly predict 3 to 5 subcellular localizations for proteins in mono- and/or diderm bacteria.

**Figure 5:**
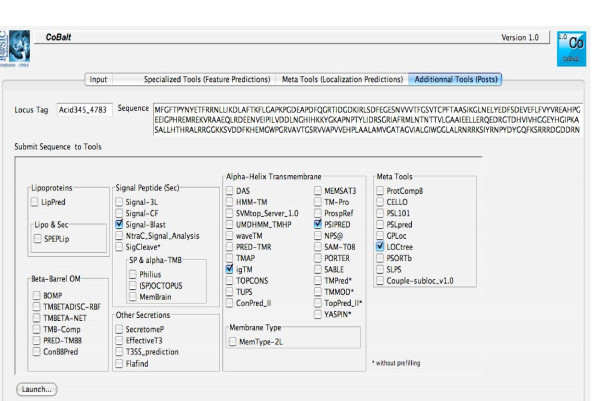
**The CoBaltDB Prefilled post window**. The "additional tools" panel enables web page submission for a set of 50 additional tools by pre-filling selected forms with selected sequence and Gram information as appropriate.

We developed a user-friendly CoBaltDB GUI as a Java 5 client application using NetBeans 5.5.1 IDE. It presents four tabs that perform specific tasks: the "input" tab (Figure 2) allows selecting the organism whose proteome localizations will be presented, using organism name completion or through an alphabetical list. Alternatively, users may also enter a subset of proteins, specified by their locus tags. The "Specialized tools" tab (Figure 3) supplies a table showing, for each protein identified by its locus tag or protein identifier, some annotation information such as its gene name, description and links to the corresponding NCBI and KEGG web pages. Clicking on a "locus tag" opens a navigator window with the related KEGG link, and clicking on a "protein Id" opens the corresponding NCBI entry web page. The table shows, for each protein and for each feature box (Tat, Sec, Lipo, αTMB, βBarrel), a heat map (white/blue) representing the percentage of tools predicting the truth/presence of the corresponding localization feature in the protein considered. Clicking on the heat map opens a new window that shows the raw data generated by each tool of the considered feature box, thus allowing the investigator to access the tool-specific information they are used to. The predictions of related feature databases are given next to the corresponding heat-map. The proteins which are referred to by the databases implemented in CobaltDB as having an experimentally determined localization appear with a yellow background colour. This representation enables the user to observe graphically the distribution of tools predicting each type of feature. The "meta-tools" tab (Figure 4) provides the predictions given by multi-modular prediction software (meta-tools or global databases) that use various techniques to predict directly three to five subcellular protein localizations in mono- and/or diderm bacteria (Table 4). The descriptions of the localizations were standardised to ease interpretation by the investigator. Both tables may be searched for occurrences of any string of characters via the search button, facilitating retrieval of a particular locus tag, protein id, accession number or even a gene name or annotation description. Both tables may be sorted with respect to any column, i.e. in alphanumerical order for the locus tags, protein identifiers, annotation descriptions and localization predictions, or in numerical order for the percentages. This makes it straightforward to identify all proteins with particular combinations of localization features. Both tables may be saved as Excel files. Finally, the CoBaltDB "additional tools" tab (Figure 5) enables queries to be submitted to a set of 50 additional tools by pre-filling the selected forms with the selected protein sequence and Gram information whenever appropriate. For this use, the investigator might have to enter additional parameters.

**Figure 6:**
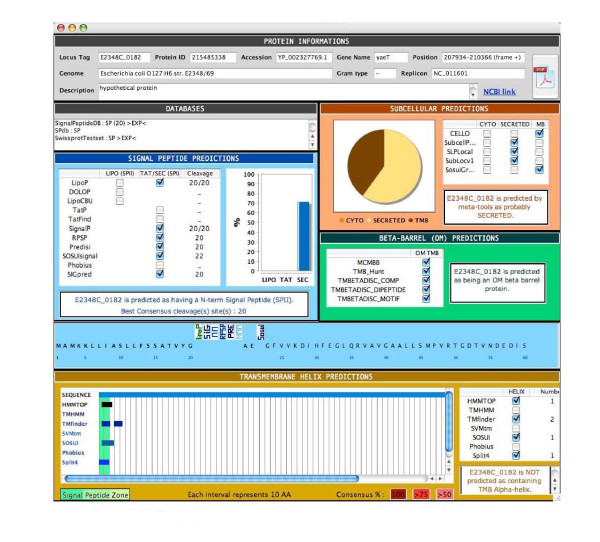
**CoBaltDB Synopsis**. For any given protein, all results are summarized in a synopsis which presents, in a unified manner, a summary of all predicted cleavage sites and membrane domains. This synopsis can be stored as a .pdf file.

Finally, for each protein, all results were summarized in a synopsis (Figure 6); the synopsis presents the results generated by all the tools in a unified manner, and includes a summary of all predicted cleavage sites and membrane domains. This "standardized" form thus provides all relevant information and lets the investigators establish their own hypotheses and conclusions. This form may be saved as a .pdf file (Figure 6). Examples of using the CoBaltDB synopsis are provided below in the second case study.

### Selected CoBaltDB uses

We propose to illustrate briefly some possible uses of CoBaltDB.

#### 1-Using CoBaltDB to compare subcellular prediction tools and databases

The various bioinformatic approaches developed for computational determination of protein subcellular localization exhibit differences in sensitivity and specificity; these differences are mainly the consequences of the types of sequences used as training models (diderms, monoderms, Archaea) and of the methods applied (regular expressions, machine learning or others). By interfacing the results from most of the reliable predictions tools, CoBaltDB provides immediate comparisons and constitutes an accurate and high-performance resource to identify and characterize candidate "non-cytoplasmic" proteins. As an example, using CoBaltDB to analyse the 82 proteins that compose the experimentally confirmed "Lipoproteome" of *E. coli K-12 *[97] shows that 72 are correctly predicted by the three precomputed tools (LipoP [59], DOLOP [57] and LIPO [56]), and that the other 10 are only identified by two of the three tools (Additional file 4A). Eight of these lipoproteins were not detected by DOLOP, because the regular expression pattern allowing detection of the lipidation sequence ([LVI] [ASTVI] [GAS] [C] lipobox) is too stringent (Additional file 4B). By comparison, the PROSITE lipobox pattern (PS00013/PDOC00013) is more permissive ([DERK](6)- [LIVMFWSTAG] (2)- [LIVMFYSTAGCQ]- [AGS]-C). This example demonstrates that using a single tool may result in errors and suggests that the best approach is to combine the various "features-based" methods available and compare their findings. This view also applies to meta-tools predictors. *E. coli *K12 lipoproteins can be found anchored to the inner or the outer membrane through attached lipid, but some of them are periplasmic (Additional file 4A). The comparison of *in silico *subcellular localization assignments with experimental findings clearly indicates that all meta-tools require significant improvements in accuracy and precision, that none should be used to the exclusion of the others. It also appears that analysis with specialized tools, organized on a "one feature at a time" basis (Lipo SPs, TAT SPs ...), most reliably gives predictions consistent with experimental data. For this purpose, CoBaltDB is a unique and innovative resource.

#### 2-Using CoBaltDB to analyse protein(s) and a proteome

**Figure 7:**
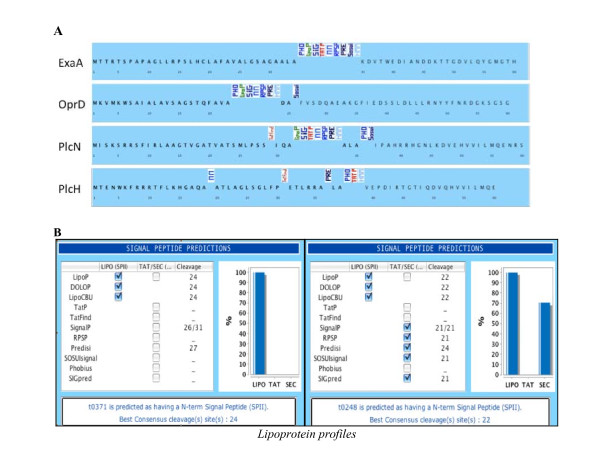
**Using CoBaltDB to analyse protein(s) and a proteome**. A: Comparative analysis of SP cleavage site predictions (proteinssecreted by *P. aeruginosa*); B: Discriminating between SPI- and SP II cleavage sites.

One valuable property of CoBaldDB is to recapitulate all pre-computed predictions in a unique A4-formated synopsis. This summary is very helpful for assessing computational data such as the variation and frequency in the predictions of signal peptide cleavage sites: such predictions are sometimes significantly consistent, but often are not in agreement with each other (Figure 7A). However, correct identification of signal peptide cleavage sites is essential in many situations, especially for producing secreted recombinant proteins.

The CoBaltDB synopsis could also be used to discriminate between SignalPeptidaseII- and SignalPeptidaseI-cleaved signals and between SPs and N-terminal transmembrane helices. Indeed, most localization predictors have difficulties distinguishing between type I and type II signal peptidase cleavages. CoBaltDB can be exploited in an interesting way to benchmark this prediction by displaying all cleavage site predictions in a "decreasing sensitivity" arrangement (SpII then Tat-dependant SPI then Sec-SPI). By considering lipoprotein datasets from different organisms, we evidenced two principal profiles (Figure 7B) and found that all experimentally validated lipoproteins score 100% (all tools give the same prediction) or 66% in the CoBaltDB LIPO column (see explanation in the paragraph above). In addition, in almost all of the examined cases, tools dedicated to Twin-arginine SP detection do not identify SpII-dependent SP, whereas the Sec-SP predictors detect both Sec and Tat-type I as well as type II signal-anchor sequences.

These observations allow us to propose, for our data set, thresholds for each box: as previously illustrated, lipoproteins have score > 66% in the LIPO prediction box; Tat-secreted proteins have 0% in the LIPO box and 100% for the two TAT-dedicated tools; Sec-secreted proteins have 33% in the LIPO Box (due to the fact that LipoP detects both SpI and SpII [59]), 0% in the TAT-tools, and > 80% in SEC-specialized tools. Rules of this type can be used to check entire proteomes for evaluation of the different secretomes as illustrated in the following case studies.

#### 3-Using CoBaltDB to compare proteomes

**Figure 8:**
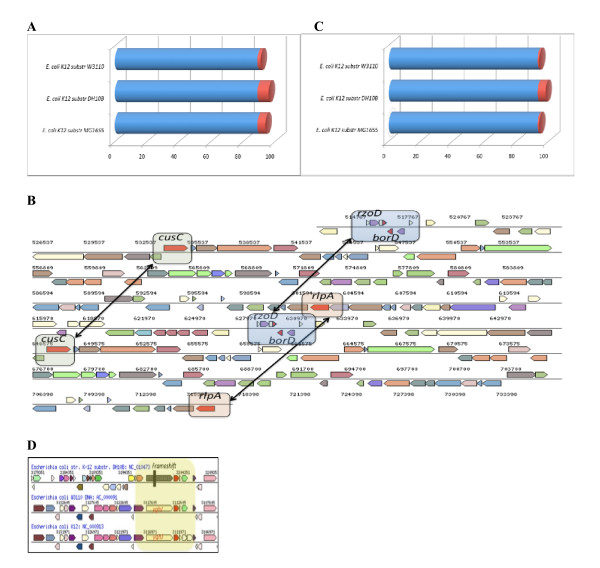
**Using CoBaltDB in comparative proteomics**. Example of E. coli K12 substrains lipoproteomes.

Using CoBaltDB and the thresholds described above, we can compare the predicted lipoproteomes (Figure 8A) of the three completely sequenced substrains of *E. coli *K12: MG1655 and W3110 (both derived from W1485 approximately 40 years ago [98]), and DH10B which was constructed by a series of genetic manipulations [99]. Each of these three substrains encode 89 lipoproteins found in both other substrains (Additional file 4). Four additional lipoproteins are detected in DH10B (BorD, CusC, RlpA and RzoD) and are second copies lipoprotein genes, present in the 113-kb tandemly repeated region of the chromosome (Figure 8B, coordinates 514341 to 627601, [99]), and strain DH10B contains one gene encoding the Rz1 proline-rich lipoprotein from bacteriophage lambda absent from the two other substrains. Lipoprotein YghJ, that shares 64% homology with *V. cholerae *virulence-associated accessory colonization factor AcfD [100], is absent from the DH10B genome annotation. However, comparative genomic analysis shows that a *yghJ *locus could be annotated in this strain but corresponds to a pseudogene caused by a frameshift event (Figure 8C). YfbK was also overlooked in the DH10B annotation process but in this case, the gene is intact. Finally, differences between lipoprotein prediction results concerning YafY, YfiM and YmbA are due to erroneous N-terminus predictions. YafY in DH10B was predicted to be a lipoprotein due to the N-terminal 17 aa-long type II signal peptide and was published as a new inner membrane lipoprotein [101]. In substrains MG1655 and WS3110, the original annotation fused the *yafY *loci with its upstream pseudogene *ykfK *(137 N-terminal aa longer). The presumed start codons of YfiM and YmbA in MG1655 were recently changed by adding 17 (*lrilfvcsllllsgcsh*) and 5 (*mkkwl*) N-terminal amino acids, respectively (PMC1325200). These modifications substantially affect the prediction of their subcellular localization. Inspection of the genomic sequences of the two other substrains leads to equivalent changes such that YfiM and YmbA in all three substrains are now predicted to be lipoproteins. In conclusion, using CoBaltDB to compare lipoproteomes between substrains, we were able to detect genomic events as well as "annotation" errors. After correction, we can conclude that the three *E. coli K12 *substrains have 93 lipoproteins in common; that one locus **whose **function is related to virulence has been transformed into a pseudogene in DH10B; and that DH10B contains five additional lipoproteins due to duplication events and to the presence of prophages absent from the other two substrains (Figure 8D).

#### 4-Using CoBaltDB to improve the classification of orthologous and paralogous proteins

**Figure 9:**
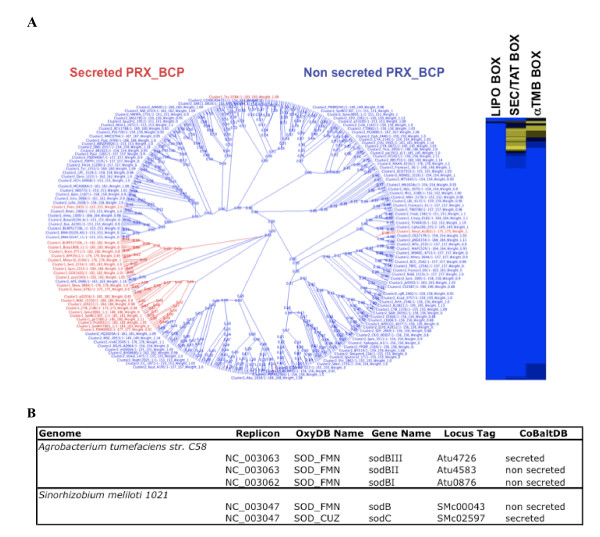
**Using CoBalt for the analysis of orthologous and paralogous proteins**. A: Phylogenetic tree of 1-cystein peroxiredoxin PRX_BCP proteins and heat map of scores in each box for each PRX_BCP protein. B: OxyGene and CoBalt predictions for SOD in *Agrobacterium tumefacins str. C58 *and *Sinorhizobium meliloti 1021*.

Protein function is generally related to its subcellular compartment, so orthologous proteins are expected, in most cases, to be in the same subcellular location. Consequently, inconsistencies of location predictions between orthologs potentially indicate distinct functional subclasses. Thus, CobaltDB can be used to help improve the functional annotation of orthologous proteins by adding the subcellular localization dimension. As an example, OxyGene, an anchor-based database of the ROS-RNS (Reactive Oxygen-Nitrogen species) detoxification subsystems for 664 complete bacterial and archaeal genomes, includes 37 detoxicifation enzyme subclasses [102]. Analysis of CoBaltDB subcellular localization information suggested the existence of additional subclasses. For example, 1-cystein peroxiredoxin, PRX_BCPs (bacterioferritin comigratory protein homologs), can be sub-divided into two new subclasses by distinguishing the secreted from the non-secreted forms (Figure 9a). Differences in the location between orthologous proteins are suggestive of functional diversity, and this is important for predictions of phenotype from the genotype.

CoBaltDB is a very useful tool for the comparison of paralogous proteins. For example, quantitative and qualitative analysis of superoxide anion detoxification subsystems using the OxyGene platform identified three iron-manganese Superoxide dismutase (SOD_FMN) in *Agrobacterium tumefaciens *but only one SOD_FMN and one copper-zinc SOD (SOD_CUZ) in *Sinorhizobium meliloti*. The number of paralogs and the class of orthologs thus differ between these two closely related genus. However, adding the subcellular localization dimension reveals that both species have machinery to detoxify superoxide anions in both the periplasm and cytoplasm: both one of the three SOD_FMN of *A. tumefaciens *and the SOD_CUZ of *S. meliloti *are secreted (Figure 9b). CoBaltDB thus helps explain the difference suggested by OxyGene with respect to the ability of the two species to detoxify superoxide.

## Discussion

CobaltDB allows biologists to improve their prediction of the subcellular localization of a protein by letting them compare the results of tools based on different methods and bringing complementary information. To facilitate the correct interpretation of the results, biologists have to keep in mind the limitations of the tools especially regarding the methodological strategies employed and the training sets used [93]. For example, most specialized tools tend to detect the presence of N-terminal signal peptides and predict cleavage sites. However the absence of an N-terminal signal peptide does not systematically indicate that the protein is not secreted. Some proteins that are translocated via the Sec system might not necessarily exhibit an N-terminal signal peptide, such as the SodA protein of *M. tuberculosis*, which is dependent on SecA2 for secretion and lacks a classical signal sequence for protein export [103]. Furthermore, there is no systematic cleavage of the N-terminal signal peptide as it can serve as a cytoplasmic membrane anchor [104,105]. Another example: although type II and type V secretion systems generally require the presence of an N-terminal signal peptide in order to utilise the sec pathway for translocation from cytoplasm to periplasm, type I and type III (and usually also type IV) systems can secrete a protein without any such signal [28,106]. Other proteins, such as Yop proteins exported by the *Yersinia *TTS system, have no classical *sec*-dependent signal sequences; however the information required to direct these proteins into the TTS pathway is contained within the N-terminal coding region of each gene [107-109].

Some challenges still need to be addressed in the prediction of the subcellular localization of proteins. For instance, bioinformatics has recently focussed on predicting proteins secreted via other pathways [110,111].

## Conclusion

We have developed CoBaltDB, the first friendly interfaced database that compiles a large number of *in silico *subcellular predictions concerning whole bacterial and archaeal proteomes. Currently, CoBaltDB allows fast access to precomputed localizations for 2,548,292 proteins in 784 proteomes. It allows combined management of the predictions of 75 feature tools and 24 global tools and databases. New specialised prediction tools, algorithms and methods are continuously released, so CoBaltDB was designed to have the flexibility to facilitate inclusion of new tools or databases as required.

In general, our analysis indicates that both feature-based and general localization tools and databases have perform diversely in terms of specificity and sensitivity; the diversity arises mainly from the different sets of proteins used during the training process and from the limitations of the mathematical and statistical methodologies applied. In all our analyses with CoBaltDB, it became clear that that the combination and comparative analysis of results of heterogeneous tools improved the computational predictions, and contributed to identifying the limitations of each tool. Therefore, CoBaltDB can serve as a reference resource to facilitate interpretation of results and to provide a benchmark for accurate and effective *in silico *predictions of the subcellular localization of proteins. We hope that it will make a significant contribution to the exploitation of *in silico *subcellular localization predictions as users can easily create small datasets and determine their own thresholds for each predicted feature (type I or II SPs for example) or proteome. This is very important, as constructing an exhaustive "experimentally validated protein location" dataset is a time-consuming process --including identifying and reading all relevant papers-- and as experimental findings about some subcellular locations are very limited.

## Availability and requirements

**Database name**: CoBaltDB

Project home page:

http://www.umr6026.univ-rennes1.fr/english/home/research/basic/software/cobalten

**Operating system(s)**: Platform independent

**Programming languages**: Java, Python and BioPython

CoBaltDB package, requirements and documentations are freely available at http://www.umr6026.univ-rennes1.fr/english/home/research/basic/software/cobalten

## Abbreviations

(AA): Amino acid; (aTMB): alpha-transmembrane; (CU): chaperone-usher; (ENP): extracellular nucleation-precipitation; (EXP): experimentally valitaded; (FEA): flagella export apparatus; (FPE): fimbrilin-protein exporter; (GUI): Graphical User Interface; (HMM): Hidden Markov Model; (LIPO): lipoprotein; (NN): Neural Network; (PRX_BCPs): bacterioferritin comigratory protein homologs; (PSSM): Position Specific Scoring Matrix; (RE): regular expression; (ROS-RNS): Reactive Oxygen-Nitrogen species; (Sec): Sec apparatus; (SOAP): Simple Object Access Protocol; (SOD_CUZ): one copper-zinc superoxide dismutase; (SOD_FMN): iron-manganese superoxide dismutase; (SP): signal peptide; (SVM): support vector machine; (T1-7SS): Type (1-7) Secretion System; (Tat): Twin-arginine translocation; (TM): transmembrane; (Wss): WXG100 secretion system.

## Authors' contributions

DG designed and implemented the CoBaltDB database and the pre-computing pipeline for automated data retrieval. SA and DG developed the user interface. CLM and FBH tested the database for functionality, and performed bioinformatics analyses leading to valuable suggestions on utility and design. CLM and SA helped coordinate the study. FBH conceived and managed the project. All authors participated in CoBaltDB design, contributed to workflow and interface designs and helped write the manuscript. All authors read and approved the final manuscript.

## Supplementary Material

Additional file 1 **List of precomputed genomes (Excel)**. A table of all complete procaryotic genomes and corresponding replicons available in CoBaltDB. Click here for file

Additional file 2 **Procaryotic subcellular localisation tools (HTML)**. This page is an inventory of all tools considered during the construction of CoBaltDB. The tools and databases related to the protein localization in procaryotic genomes are sorted by type of prediction. For each tool, a short description and the corresponding web link are displayed. Click here for file

Additional file 3 **Monoderm and Diderm classification of genomes (PNG)**. Picture showing the cellular organization type (monoderm or diderm) for phylum in CoBaltDB. Click here for file

Additional file 4 **Using CoBalt in comparative proteomics (PDF)**. Example of the lipoproteomes *of E. coli K12 *substrains, experimentally confirmed by EcoGene. Table1A: Prediction results for the 89 confirmed lipoproteins in the three substrains DH10B, MG1655 et W3110. Table1B: The lipoproteins that are not recognized by DOLOP have a sequence which does not match the DOLOP lipoBox pattern [LVI] [ASTVI] [ASG] [C]. Click here for file
