# Supplementary material for: CoBaltDB: Complete bacterial and archaeal orfeomes subcellular localization database and associated resources
Source: BMC Microbiol. 2010 Mar 23;10:88. doi: 10.1186/1471-2180-10-88 (PMC2850352; doi:10.1186/1471-2180-10-88)
Supplement: Additional file 2 — Procaryotic subcellular localisation tools (HTML). This page is an inventory of all tools considered during the construction of CoBaltDB. The tools and databases related to the protein localization in procaryotic genomes are sorted by type of prediction. For each tool, a short description and the corresponding web link are displayed. [file 1471-2180-10-88-S2.PDF]

COBALT_DB:

Complete Bacterial and Archaeal Orfeomes Subcellular Localization Database

Prokaryotic localization tools and databases

CoBaltDB (in submission):
Biologists are confronting to a very large number of computational tools that target various localization features, using different methods, with dissimilar specificity and sensitivity. As a result, exploiting these computer resources to accurately predict protein localization involves to query all tools and to compare every prediction outputs, which is a fastidious task. For this reason, we developed CoBalt_DB, a comprehensive database that friendly interface all prediction outputs for complete prokaryotic proteomes.

For CoBaltDB construction we have analysed a very large number of tools and databases related to prokaryotic localization prediction. The following list of tools and databases wants to be the most exhaustive as possible.

● Tools and databases present in CoBaltDB ○ Others Tools and databases

---

SIGNAL PEPTIDES PREDICTION

Type I Signal Peptides - TAT

● TATFIND 1.4 *(Rose et al., 2002) [PMID:12180915]* Regular expression

TATFIND 1.4 predicts the presence of prokaryotic Twin-Arginine Translocation (Tat) signal peptides.

● TatP 1.0 Server *(Bendtsen et al., 2005) [PMID:15992409]* Regular expression + Neural Network (NN)

TatP 1.0 server predicts the presence and location of Twin-arginine signal peptide cleavage sites in bacteria. The method incorporates a prediction of cleavage sites and a signal peptide/non-signal peptide prediction based on a combination of two artificial neural networks.

Type I Signal Peptides - SEC

● PrediSi *(Hiller et al., 2004) [PMID:15215414]* Position weight matrix

PrediSi (PREDIction of SIgnal peptides) is a software tool for predicting signal peptide sequences and their cleavage positions in bacterial and eukaryotic proteins.

● PRED-SIGNAL *(Bagos et al., 2009) [PMID:18988691]* Hidden Markov Model (HMM)

Prediction of Signal Peptides in Archaea with Hidden Markov Models.

● Rapid Prediction of Signal Peptides - RPSP *(Plewczynski et al., 2008) [PMID:18506221]* Neural Network (NN)

The RPSP program detects signal peptides in proteins. The method is based on neural networks trained on short sequence fragments of proteins extracted from the Swiss-Prot database. The RPSP is able to deal separately with prokaryotic and eukaryotic sequences.

● Sigcleave Von Heijne method

Uses the method of von Heijne as modified by von Heijne in his later book where treatment of positions -1 and -3 in the matrix is slightly altered.

● Signal-Blast *(Frank et al., 2008) [PMID:18697773]* BLASTP

High-performance signal peptide prediction based on sequence alignment techniques.

● Signal3L *(Shen and Chou 2007) [PMID:17880924]* Multi program modules

Signal-3L is an automated method for predicting signal peptide sequences and their cleavage sites in eukaryotic and bacterial protein sequences. It consists of three prediction engines working respectively for the

following three progressively deepening layers:#(1) Identifying a query protein as secretory or non-secretory by an ensemble classifier;#(2) Selecting a set of candidates for the possible signal peptide cleavage sites of a query secretory protein by a subsite-coupled discrimination algorithm;#(3) Determining the final cleavage site by fusing the global sequence alignment outcome for each of the aforementioned candidates through a voting system.

- SignalCF *(Chou et al., 2007) [PMID:17434148]* Multi program modules

Signal-CF is an automated method for predicting signal peptide sequences and their cleavage sites in eukaryotic and bacterial protein sequences. It is a 2-layer predictor: the 1st-layer prediction engine is to identify a query protein as secretory or non-secretory; if it is secretory, the process will be automatically continued with the 2nd-layer prediction engine to further identify the cleavage site of its signal peptide.

- SignalP 3.0 Server *(Bendtsen et al., 2004) [PMID:15223320 / 9051728 / 9783217]* Hidden Markov Model (HMM) + Neural Network (NN)

SignalP 3.0 server predicts the presence and location of signal peptide cleavage sites in amino acid sequences from different organisms: Gram-positive prokaryotes, Gram-negative prokaryotes, and eukaryotes. The method incorporates a prediction of cleavage sites and a signal peptide/non-signal peptide prediction based on a combination of several artificial neural networks and hidden Markov models.

- SIG-Pred *(J.R. Bradford, University of Leeds)* Matrix

Signal Peptide Prediction

- SOSUIsignal *(Gomi et al., 2004)* Multi program modules

The tripartite structure is recognized by 3 modules of the software systeM. The first module numerates hydrophobic segment in N-terminal 100 residues, the second predicts signal sequences including both signal peptides and signal anchors, and the third discriminates signal peptide.

Type II (Lipoprotein) Signal Peptides

- DOLOP *(Babu et al., 2006) [PMID:16585737]* Regular expression

Database of bacterial lipoproteins and also a predictive algorithm

- LIPO *(Berven et al., 2006) [PMID:16311759]* Regular expression

Lipo can analyse polypeptide sequences encoded within the genome of Gram-negative bacteria and report the sequences with a possible lipo-box. The lipo-box is recognised on the basis of the critera given by Lipo.

- LipPred *(Taylor et al., 2006) [PMID:17597883]* Naive Bayesian Network

A probabilistic sequence model was used to allow for lipobox sequence variations. A sequence model of 10 residues, consisting of the conserved clevage site cysteine and the 9 residues previous to it, is used. This model is tested against a Naive-Bayesian network to test whether or not the query protein is a lipoprotein.

- PRED-LIPO *(Bagos et al., 2008) [PMID:19367716]* Hidden Markov Model (HMM)

Prediction of Lipoprotein and Secretory Signal Peptides in Gram-positive Bacteria with Hidden Markov Models.

- SpLip *(Setubal et al., 2006)[PMID:16385121]* Weight matrix

Lipoprotein computational prediction in spirochaetal genomes.

Type I and type II Signal Peptides

- SPEPLip *(Fariselli et al., 2003)* Neural Network (NN)

A Neural-Network-based system is trained and tested on a set of well annotated proteins to tackle the problem of predicting the signal peptide in protein sequences.

Type III Signal Peptides

- FlaFind (archaeal type III + bacteria type IV pilin-like) *(Szabó et al., 2007) [PMID:17114255]* Amino Acid features

A sequence is FlaFind positive if (i) the sequence has one or two TMHMM-predicted hydrophobic segments, (ii) the first hydrophobic segment begins within the first 30 amino acids of the protein sequence, and (iii) the pattern [KR][GA][ALIFQMVED][ILMVTAS] is found preceding the hydrophobic segment but not more than 10 amino acids away from the beginning of the hydrophobic segment.

- EffectiveT3 *(Arnold et al., 2007) [PMID:19390696]* Machine learning

  Sequence based prediction of Type III secreted proteins.

- T3SS *(Loewer et al., 2009) [PMID:19526054]* Hidden Markov Model (HMM) + Neural Network (NN)

  Type III secretion system effector prediction (T3SS) predicts the existence of type III secretion system (T3SS) signals in amino acid sequences.

Long Signal Peptides

- NtraC Siganl Analysis *(Hiss et al., 2008) [PMID:18648515]* Amino Acid features

  We discovered a two-domain organization ("NtraC model") in many long signals from vertebrate precursor proteins.

Signal Peptide Variants

- SecretomeP 2.0 Server *(Bendtsen et al., 2005) [PMID:16212653]* Pattern and artificial Neural Network (NN).

  The SecretomeP 2.0 server produces ab initio predictions of non-classical i.e. not signal peptide triggered protein secretion. The method queries a large number of other feature prediction servers to obtain information on various post-translational and localizational aspects of the protein, which are integrated into the final secretion prediction.

MULTI-FEATURES PREDICTION

Type I Signal Peptides + Transmembrane Alpha-Helices

- MemBrain *(Shen et al., 2008) [PMID:18545655]* Machine-learning

  A machine-learning based predictor, MemBrain, which integrates a number of modern bioinformatics approaches including sequence representation by multiple sequence alignment matrix, the optimized evidence-theoretic K-nearest neighbor prediction algorithm, fusion of multiple prediction window sizes, and classification by dynamic threshold.

- Philius *(Reynolds et al., 2008) [PMID:18989393]* Hidden Markov Model (HMM)

  It is inspired by a previously published HMM, Phobius, and combines a signal peptide submodel with a transmembrane submodel. We introduce a two-stage DBN decoder that combines the power of posterior decoding with the grammar constraints of Viterbi-style decoding. Philius also provides protein type, segment, and topology confidence metrics to aid in the interpretation of the predictions.

- Phobius *(Käll et al., 2004 / Käll et al., 2007) [PMID:15111065 / 17483518]* Hidden Markov Model (HMM)

  A combined transmembrane topology and signal peptide predictor.

- OCTOPUS / SPOCTOPUS *(Viklund et al., 2008) [PMID:18474507 / 18945683]* BLAST homology + Neural Network (NN) + Hidden Markov Model (HMM)

  Prediction of membrane protein topology and signal peptides.

- TUPS *(Zhou et al., 2005) [PMID:15980453]* Consensus Tools

  Combines the prediction of THUMBUP and UMDHMM-TMHP for TM segments and PHOBIUS for the identification of signal peptides.

Type I and II Signal Peptides + Transmembrane Alpha-Helices

- LipoP 1.0 Server *(Juncker et al., 2003) [PMID:12876315]* Hidden Markov Model (HMM) + Neural Network (NN)

  The LipoP 1.0 server produces predictions of lipoproteins and discriminates between lipoprotein signal peptides, other signal peptides and n-terminal membrane helices in Gram negative bacteria.

MEMBRANE PROTEIN PREDICTION

Transmembrane Alpha-Helices

- BPROMPT *(Taylor et al., 2003)[PMID:12824397]* Bayesian Belief Network (consensus)
  Using a Bayesian Belief Network to combine the results of other prediction methods.

- DAS *(Cserzo et al., 1997) [PMID:9278280]* Dense Alignment Surface (DAS) method
  The DAS server will predict transmembrane regions of a query sequence.

- HMMTOP *(Tunasdy and Simon, 2001) [PMID:11590105]* Hidden Markov Model (HMM)
  HMMTOP is an automatic server for predicting transmembrane helices and topology of proteins.

- igTM *(Peris et al., 2008) [PMID:18783592]* Grammatical Inference
  Using Grammatical Inference (GI) to localize transmembrane segments. The GI process is based specifically on the inference of Even Linear Languages.

- MEMSAT3 *(Jones et al., 1994 / Jones et al., 2007) [PMID:8130217 / 17237066]* Neural Network (NN)
  MEMSAT3 is a program which predicts the secondary structure and topology of all-helix integral membrane proteins based on the recognition of topological models.

- HMM-TM *(Bagos et al., 2006) [PMID:16597327]* Hidden Markov Model (HMM)
  Prediction of Transmembrane Alpha-Helical Proteins.

- orienTM *(Liakopoulos et al., 2001)[PMID:11477216]* SwissProt Statistical analysis
  It uses position-specific statistical information for amino acid residues which belong to putative non-transmembrane segments derived from statistical analysis of non-transmembrane regions of membrane proteins stored in the SwissProt database.

- PRED-TMR2 *(Pasquier et al., 1999)[PMID:10469822]* NN
  The algorithm refines a standard hydrophobicity analysis with a detection of potential termini ("edges", starts and ends) of transmembrane regions. A extension with a pre-processing stage represented by an artificial neural network to discriminate with a high accuracy transmembrane proteins from soluble or fibrous ones.

- PRED-TMR *(Pasquier et al., 1999) [PMID:10360978]* Amino Acid features
  The algorithm refines a standard hydrophobicity analysis with a detection of potential termini ("edges", starts and ends) of transmembrane regions.

- SOSUI *(Mitaku et al., 2002) [PMID:12016058]* Amino Acid features
  System for membrane protein discrimination and transmembrane helix prediction.

- S_TMHMM *(Viklund and Elofsson, 2004)[PMID:15215532]* HMM
  Retrained version of TMHMM.

- SVMtm *(Yuan et al., 2004) [PMID:14978706]* SVM
  SVMtm is a support vector machine-based transmembrane (TM) helices predictor.

- SVMtop Server 1.0 *(Lo et al., 2008) [PMID:18081245]* SVM
  A Support Vector Machine-based method for transmembrane helix and topology prediction.

- TMAP *(Persson et al., 1997) [PMID:9246628]* Amino Acid features
  TMAP: a new email and WWW service for membrane-protein structural predictions.

- TM-Finder *(Deber et al., 2001) [PMID:11266608]* Amino Acid features

The TM-Finder is a Trans-Membrane protein prediction tool that was developed based on an algorithm, data, and prototype program produced by Dr. Charles Deber and his research team. The program shows the regions predicted to be trans-membrane based on the helicity and hydrophobicity of the neighbouring amino acid sequences.

- TMHMM Server v. 2.0 *(Krogh et al., 2001) [PMID:11152613]* Hidden Markov Model (HMM)

  Prediction of transmembrane helices in proteins.

- TMMOD *(Kahsay et al., 2005) [PMID:15691854]* Hidden Markov Model (HMM)

  TMMOD is a server for transmembrane proteins topology prediction using a hidden Markov model. TMMOD uses TMHMM as a prototype, but differs from TMHMM by the architecture of the submodels for loops on both sides of the membrane and also by the model training procedure.

- TMPred Weight-matrices

  The algorithm is based on the statistical analysis of TMbase, a database of naturally occuring transmembrane proteins. The prediction is made using a combination of several weight-matrices for scoring.

- TM Pro *(Ganapathiraju et al., 2008 / Ganapathiraju et al., 2007) [PMID:18315857 / 17724062]* AA features

  Transmembrane helix prediction using amino acid property features.

- TOPCONS *(Bernsel et al., 2009) [PMID:19429891]* Tools Consensus

  Consensus prediction of membrane protein topology. Using SCAMPI (single sequence mode), SCAMPI (multiple sequence mode), PRODIV-TMHMM, PRO-TMHMM and OCTOPUS.

- TopPred II *(Claros et al., 1994) [PMID:7704669]* -

  -

- TransMem *(Aloy et al., 1997)[PMID:9183525]* NN

  TransMem, based on a neural network and running on personal computers (either Apple Macintosh or PC, using Excel worksheets), for the prediction and distribution of amino acid residues in transmembrane segments of integral membrane proteins is reported.

- UMDHMM TMHP *(Zhou et al., 2003) [PMID:12824500]* Hidden Markov Model (HMM)

  University of Maryland Hidden Markov Model for topology prediction of trans membrane-helical- protein.

- waveTM *(Pashou et al., 2004) [PMID:15107018]* Algorithm (hydropathy signals)

  Dynamic programming algorithm on wavelet-denoised 'hydropathy' signals.Prediction of transmembrane segments in proteins.

- YASPIN *(lin et al., 2005) [PMID:15377504]* Hidden Neural Network

  ASPIN is a HNN (Hidden Neural Network) secondary structure prediction program that uses the PSI-BLAST algorithm to produce a PSSM for the input sequence, which it then uses to perform its prediction.

Transmembrane Topology

- APSSP2 *(Raghava G. P. S., 2002)* NN + PSIBLAST + Example based learning technique

  It uses the standard neural network and multiple sequence alignment generated by PSIBLAST instead of single sequence. In second step it predicts the secondary of proteins using modified example based learning (EBL) technique. In third step secondary structure predicted from above two steps are combined in order to predict the final structure.

- ConPred II *(Arai et al., 2004) [PMID:15215417]* Tools Consensus

  The prediction methods used in ConPred II are KKD, TMpred, TopPred II, DAS, TMAP, MEMSAT 1.8, SOSUI, TMHMM 2.0 and HMMTOP 2.0.

- NPS@ *(Combet et al., 2000) [PMID:10694887]* Tools Consensus

  Multi tools consensus prediction.

- PRALINE_TM *(Simossis et al., 2005)[PMID:15980472]* Alignement + Consensus Tools

A multiple sequence alignment toolbox that integrates homology-extended and secondary structure information.

- PRODIV-TMHMM webwerver *(Viklund et al., 2004)[PMID:15215532]* Multi Hidden Markov Model (HMMs)

The PRODIV-TMHMM webserver actually consists of four separate topology predictors. PRODIV-TMHMM, PRO-TMHMM and S-TMHMM are hidden Markov model based servers for topology prediction of transmembrane proteins.

- ProspRef Knowledge-based method

A Knowledge-based method for predicting essential protein secondary structures

- PSIPRED v2.6 *(Jones, 1999 / Bryson et al., 2005) [PMID:10493868 / 15980489]* Neural Network (NN), position-specific scoring matrices

A two-stage neural network has been used to predict protein secondary structure based on the position specific scoring matrices generated by PSI-BLAST.

- SABLE *(Adamczak et al., 2005) [PMID:15768403]* Neural Network (NN)

Accurate sequence-based prediction of relative Solvent AccessiBiLitiEs, secondary structures and transmembrane domains for proteins of unknown structure

- SAM-T08 *(Karplus, 2009) [PMID:19483096]* Hidden Markov Model (HMM)

Finds similar protein sequences in NR and aligns them, providing sequence logos that show relative conservation of different positions. Local structure predictions are done with neural nets for several different local structure alphabets, and hidden Markov models are created. Fold recognition and alignment to proteins in the Protein Data Bank are done, and a full three-dimensional model is constructed.

- Split 4.0 Server *(Juretic et al., 2002) [PMID:12086524]* Amino Acid features with preference functions

Automatic selection of optimal amino acid attribute and corresponding preference functions. The best topological model is selected by choosing the highest absolute bias parameter that combines the bias in basic charge motifs and the bias in positive residues (the "positive inside rule") with the charge difference across the first transmembrane segment.

Transmembrane Beta-Barrel

- BOMP *(Berven et al., 2004) [PMID:15215418]* Pattern, Amino Acid features

Based on two separate components to recognize integral beta-barrel proteins. The first component is a C-terminal pattern typical of many integral beta-barrel proteins. The second component calculates an integral beta-barrel score of the sequence based on the extent to which the sequence contains stretches of amino acids typical of transmembrane beta-strands.

- ConBBPRED *(Bagos et al., 2005) [PMID:15647112]* Consensus Tools

Consensus prediction with PRED-TMBB, TM-BETA, B2TMPRED, BETATM, HMM-B2TMR, ProfTMB, TBBpred, PSIPRED.

- MCMBB *(Bagos et al., 2004)* Hidden Markov Model (HMM)

MCMBB is a fast algorithm, which discriminates beta-barrel outer membrane proteins from globular proteins and from alpha-helical membrane proteins. The algorithm is based on a 1st order Markov Chain model, which captures the alternating pattern of hydrophilic-hydrophobic residues occurring in the membrane-spanning beta-strands of beta-barrel outer membrane proteins.

- PRED-TMBB *(Bagos et al., 2004) [PMID:15215419]* Hidden Markov Model (HMM)

The method is based on a Hidden Markov Model, trained according to the Conditional Maximum Likelihood criterion

- TMB-Comp SVM classifier

Screen sequence sets for potential transmembrane beta strands and discriminate between TMB/non-TMB proteins using a series of composition based predictors with results fed into a support vector machine (SVM) classifier.

- TMBETADISC_COMP *(Gromiha et al., 2005) [PMID:15980447]* Amino Acid features

Discriminating outer membrane proteins from other folding types of globular and membrane proteins is an important task both for dissecting outer membrane proteins (OMPs) from genomic sequences and for the successful prediction of their secondary and tertiary structures.

- TMBETADISC_DIPEPTIDE *(Gromiha et al., 2005) [PMID:15980447]* Dipeptide composition

Discriminating outer membrane proteins from other folding types of globular and membrane proteins is an important task both for dissecting outer membrane proteins (OMPs) from genomic sequences and for the successful prediction of their secondary and tertiary structures.

- TMBETADISC_MOTIF *(Gromiha et al., 2005) [PMID:15980447]* Motif

Discriminating outer membrane proteins from other folding types of globular and membrane proteins is an important task both for dissecting outer membrane proteins (OMPs) from genomic sequences and for the successful prediction of their secondary and tertiary structures.

- TMBETADISC-RBF *(Ou et al., 2008) [PMID:18434251]* Radial basis function network, PSSM

Method based on radial basis function networks and position specific scoring matrix (PSSM) profiles generated by PSI-BLAST and non-redundant protein database.

- TMBETA-NET *(Gromiha et al., 2005) [PMID:15980447]* Amino Acid features

This program discriminates outer membrane proteins and predicts transmembrane beta strands in an outer membrane protein from its amino acid sequence

- TMBETA-SVM *(Park et al., 2005)[PMID:16204348]* SVM

Method based on support vector machines using amino acid composition and residue pair information.

- TMB-HMM *(Andrew Garrow)* Hidden Markov Model (HMM) + Support Vector Machine (SVM)

Screen sequence sets for potential transmembrane beta strands and discriminate between TMB/non-TMB proteins using a hidden Markov model (HMM) and support vector machine (SVM) based approach.

- TMB-Hunt2 *(Garrow et al., 2005) [PMID:15980452]* Amino Acid features and Hidden Markov Model (HMM) into SVM classifier

Screen sequence sets for potential transmembrane beta strands and discriminate between TMB/non-TMB proteins using a series of composition and HMM based predictors with results fed into a support vector machine (SVM) classifier.

Membranes Proteins Types

- MemType-2L *(Chou et al., 2007) [PMID:17586467]* Pse-PSSM, OET-KNN ensemble classifier

MemType-2L is featured by incorporating the evolution information through representing the protein samples with the Pse-PSSM (Pseudo Position-Specific Score Matrix) vectors, and by containing an ensemble classifier formed by fusing many powerful individual OET-KNN (Optimized Evidence-Theoretic K-Nearest Neighbor) classifiers.

Cell-Wall Anchored Proteins

- CW-Pred *(Litou et al., 2008) [PMID:18464329]* Hidden Markov Model (HMM) on motif

Prediction of Cell Wall-Anchored Proteins in Grampositive Bacteria. A HMM approach for predicting the LPXTG-anchored cell wall proteins of Gram-positive bacteria was developed and compared against existing methods.

Protein Secondary Structure

- PORTER *(Pollastri et al., 2005) [PMID:15585524]* Neural Network (NN)

Porter is a server for protein secondary structure prediction based on an ensemble of 45 BRNNs (bidirectional recurrent neural networks).

SUBCELLULAR LOCALIZATION PREDICTION (multi features)

CELLO v.2.5 *(Yu et al., 2006) [PMID:16752418]* Multi-class Support Vector Machine (SVM) classification system

CELLO is a multi-class SVM classification system. CELLO uses 4 types of sequence coding schemes: the amino acid composition, the di-peptide composition, the partitioned amino acid composition and the sequence composition based on the physico-chemical properties of amino acids.

couple-subloc v1.0 Amino Acid features

couple-subloc is a prediction system for protein subcellular localization based on amino acid composition alone.

Gneg-Ploc *(Chou et al., 2006) [PMID:17137343]* Basic classifiers

Featured by fusing many basic classifiers each being trained with a stringent data set containing proteins with strictly less than 25% sequence identity to one another in a same location group

Gpos-Ploc *(Shen et al., 2007) [PMID:17244638]* Basic classifiers

Featured by fusing many basic classifiers, each of which was engineered according to the optimized evidence-theoretic K-nearest neighbors rule.

LOCtree *(Nair et al., 2005) [PMID:15808855]* SVM

LOCtree is a novel system of support vector machines (SVMs) that predict the subcellular localization of proteins, and DNA-binding propensity for nuclear proteins, by incorporating a hierarchical ontology of localization classes modeled onto biological processing pathways.

PredictProtein *(Rost et al., 2004)[PMID:15215403]* Aligenement + Multi methods

PredictProtein retrieves similar sequences in the database and predicts aspects of protein structure and function.

ProtCompB *(SoftBerry)* Multi-methods

ProtCompB combines several methods of protein localization prediction - Linear Discriminant Function-based prediction; direct comparison with bases of homologous proteins of known localization; comparisons of pentamer distributions calculated for query and DB sequences; prediction of certain functional peptide sequences, such as signal peptides and transmembrane segments.

Proteome Analyst - Subcell Specialization Server 2.5 *(Lu et al., 2004) [PMID:14990451]* Multiple classifiers

The Proteome Analyst Specialized Subcellular Localization Server (PA-SUB) is part of Proteome Analyst (PA). PA is a web server built to predict protein properties, such as general function, in a high-throughput fashion. PA-SUB is specialized to predict the subcellular localization of proteins using established machine learning techniques.

PROTEUS2 *(Liakopoulos et al., 2008)[PMID:18483082]* Multi-methods

Using a combination of progressive multi-sequence alignment, structure-based mapping, hidden Markov models, multi-component neural nets and up-to-date databases of known secondary structure assignments

PSL101 1.0 Server *(Su et al., 2007) [PMID:17825110]* SVM (one-VS-one SVM) + structure homology approach

A protein subcellular localization prediction method based on compartment-specific features and structure conservation.

PSLpred *(Bhasin et al., 2005) [PMID:15699023]* SVM

SLpred is a hybrid approach-based method that integrates PSI-BLAST and three SVM modules based on compositions of residues, dipeptides and physico-chemical properties.

PSORTb *(Rey et al., 2004) [PMID:15608169]* Multi program modules

PSORTb v.2.0 consists of multiple analytical modules, each of which analyzes one biological feature known to influence or be characteristic of subcellular localization. The modules may act as a binary predictor, classifying a protein as either belonging or not belonging to a particular localization site, or they may be multi-category, able to assign a protein to one of several localization sites.

SLP-Local *(Matsuda et al., 2005) [PMID:16251364]* Amino Acid features

Subcellular Location Predictor based on Local features of amino acid sequence

- SLPS *(Jia et al., 2007) [PMID:17428441]* Nearest Neighbor

  Nearest Neighbor Algorithm incooperated with functional domain composition approach.

- SosuiGramN *(Imai et al., 2008) [PMID:18795116]* Physicochemical parameters

  It uses only physicochemical parameters of the N- and C-terminal signal sequences, and the total sequence.

- SubcellPredict *(Niu et al., 2008) [PMID:18506593]* Algorithm (Amino Acid composition)

  The method of subcellular location type prediction provided in its web-site is based on the AdaBoost Learner Algorithm incorporated with amino acid composition.

- SubLoc v1.0 *(Hua and Sun, 2001) [PMID:11524373]* SVM

  Support Vector Machine has been introduced to predict the subcellular localization of proteins from their amino acid compositions (3 subcellular locations in prokar yotic organisms)

- Tbpred *(Rashid et al., 2007) [PMID:17854501]* SVM

  In order to utilize evolutionary information, a SVM model was developed using PSSM (Position-Specific Scoring Matrix) profiles obtained from PSI-BLAST (Position-Specific Iterated BLAST) and overall accuracy achieved was of 86.62% with average accuracy of 73.71%. In addition, HMM (Hidden Markov Model), MEME/MAST (Multiple Em for Motif Elicitation/Motif Alignment and Search Tool) and hybrid model that combined two or more models were also developed.

DATABASES

Signal Peptides Databases

- EXProt *(Saleh et al., 2001) [PMID:11376951]* -

  ExProt (M.T. Saleh et al. 2001 Gene 269:195-204) is a program designed to identify exported proteins targeted through the Sec-pathway. It looks for Sec-dependent signal sequences within the N-terminal 45 amino acids. It is capable of discriminating between signal peptidase I cleavage sites (secreted proteins) and signal peptidase II sites (lipoproteins).

- Signal Peptide DB -

  Based of the UniProt Knowledgebase Release 14.7 consisting of UniProtKB/Swiss-Prot Release 56.7 and UniProtKB/TrEMBL Release 39.7 (January 20, 2009). In both cases, sequence entries were extracted that contain SIGNAL PEPTIDE as key name within the FT line (Feature table data).

- SPdb *(Choo et al., 2005) [PMID:16221310]* Keyword search

  SPdb is a signal peptide database containing signal sequences of archaea, prokaryotes and eukaryotes.

- Swissprot signal testset *(Menne et al., 2000) [PMID:11099261]* -

  Experimentally verified signal peptide dataset

Subcellular Localization Databases

- Augur DB *(Billion et al., 2006) [PMID:16966358]* Multi program modules

  An automatic prediction pipeline that integrates major surface prediction algorithms and enables comparative analysis, classification and visualization for gram-positive bacteria on a genomic scale.

- DBMLoc *(Zhang et al., 2008)[PMID:18304364]* -

  DBMLoc collect proteins with multiple subcellular localization annotations collected from primary protein databases, specific subcellular localization databases and literature texts.

- EchoLOCATION *(Horler et al., 2009) [PMID:19015139]* -

  EchoLOCATION is our comprehensive subcellular location analysis for all E. coli proteins, created using the publicly available prediction algorithms together with experimental data and in-house manual curation.

LocateP DataBase *(Zhou et al., 2008) [PMID:18371216]* Multi-SCL

LocateP combines many of the existing high-precision protein subcellular-location (SCL) predictors identifiers with its own newly developed identifiers for specific SCLs.

LOCTARGET *(Nair et al., 2004)[PMID:15215440]* Multi-methods

Subcellular localization is currently predicted using four different methods: predictNLS (nuclear localization signal), LOChom (using homology), LOCkey (using keywords) and LOCtree (prediction based on hierarchical support vector machines). The reported localization is based on the method which predicts localization of a given protein with the highest confidence.

PSORTdb *(Rey et al., 2004) [PMID:15608169]* Multi program modules

A database of protein subcellular localizations for bacteria, divided in a computational dataset (cPSORTdb) and a experimentally confirmed dataset (ePSORTdb).

Transmembrane Databases

ecce Multi program modules

The E. coli Cell Enveloppe Protein database (ecce) is a functional classification of cell envelope proteins.

Hhomp *(Remmert et al., 2009) [PMID:19429691]* Hidden Markov Model (HMM)

Homology detection of OuterMembrane Proteins (OMPs) by HMM-HMM comparison

MPtopo *(Jayasinghe et al., 2001)[PMID:11266632]* -

A database of Membrane Proteins whose topologies have been verified experimentally by means of crystallography, gene fusion, and other methods.

Orientations of Proteins in Membranes (OPM) database *(Lomize et al., 2006)[PMID:16397007]* Multi-methods

The database provides a collection of transmembrane, monotopic and peripheral proteins from the Protein Data Bank whose spatial arrangements in the lipid bilayer have been calculated theoretically and compared with experimental data. The database allows analysis, sorting and searching of membrane proteins based on their structural classification, species, destination membrane, numbers of transmembrane segments and subunits, numbers of secondary structures and the calculated hydrophobic thickness or tilt angle with respect to the bilayer normal.

PDBTM *(Tusnády et al., 2005)[PMID:15608195]* TMDET algorithm

It aims to collect all transmembrane proteins that are deposited in the protein structure database (PDB) and to determine their membrane-spanning regions. These assignments are based on the TMDET algorithm, which uses only structural information to locate the most likely position of the lipid bilayer and to distinguish between transmembrane and globular proteins.

PROFtmb *(Bigelow et al., 2004) [PMID:15141026]* -

This service predicts whole-protein class (TMB/non-TMB) by providing a length-based z-score for a given protein.

THGS DB *(Fernando et al., 2003) [PMID:14681375]* Motif

A web based database of Transmembrane Helices in Genome Sequences

TMBC-Database -

Transmembrane Barrel Classification Database

TMBETA-GENOME *(Gromiha et al., 2007)[PMID:17088282]* Multi-methods

Collection of amino acid sequences for all the completed genomes and the annotated trans beta-barrel membrane proteins (TMBs) using different discrimination algorithms.

TMPDB *(Ikeda et al., 2003) [PMID:12520035]* -

Transmembrane Proteins with Experimentally-Characterized Transmembrane Topologies

- TOP_DB *(Tusnády et al., 2008) [PMID:17921502]* Inclut PDBTM

The database collects the details of various experiments carried out to learn about the topology of particular transmembrane proteins. In addition to literature-derived data, an extensive collection of structural data was also compiled from Protein Data Bank (PDB) and from Protein Data Bank of Transmembrane Proteins (PDBTM) by utilising the TMDET algorithm. For each protein the most probable topology consistent with the collected experimental constraints was also calculated using HMMTOP transmembrane topology prediction algorithm.

Others Databases

- DTTSS -

Database of Type III Secretion System

- EcoProDB *(Yun et al., 2007)[PMID:17623702]* Identification on 2D gels

The database contains information on E. coli proteins identified on 2D gels along with other resources collected from various databases and published literature, with a special feature of showing the expression levels of E. coli proteins under different genetic and environmental conditions.
