# Supplementary figures and images for: CoBaltDB: Complete bacterial and archaeal orfeomes subcellular localization database and associated resources
Source: BMC Microbiol. 2010 Mar 23;10:88. doi: 10.1186/1471-2180-10-88 (PMC2850352; doi:10.1186/1471-2180-10-88)

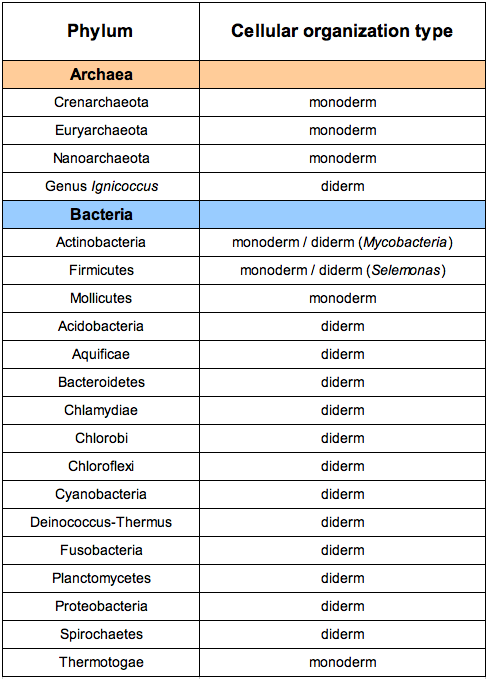

Supplement: Additional file 3 — Monoderm and Diderm classification of genomes (PNG). Picture showing the cellular organization type (monoderm or diderm) for phylum in CoBaltDB. [file 1471-2180-10-88-S3.PNG]
