# Supplementary material for: CoBaltDB: Complete bacterial and archaeal orfeomes subcellular localization database and associated resources
Source: BMC Microbiol. 2010 Mar 23;10:88. doi: 10.1186/1471-2180-10-88 (PMC2850352; doi:10.1186/1471-2180-10-88)
Supplement: Additional file 4 — Using CoBalt in comparative proteomics (PDF). Example of the lipoproteomes of E. coli K12 substrains, experimentally confirmed by EcoGene. Table1A: Prediction results for the 89 confirmed lipoproteins in the three substrains DH10B, MG1655 et W3110. Table1B: The lipoproteins that are not recognized by DOLOP have a sequence which does not match the DOLOP lipoBox pattern [LVI] [ASTVI] [ASG] [C]. [file 1471-2180-10-88-S4.PDF]

Table 1A											
	LipoP	DOLOP	LipoCB	OM box	Eperimental localization	PSORTb	PASUB	ProtCompB	SLP	SubLo	SosuiGramN
acrA	☑	☑	☑	☑	**P [15155734]**	M	IM	P	P	P	OM
acrE	☑	☑	☑	☐	**P [1720861]**	M	IM	P	P	P	OM
amiD	☑	☑	☑	☐	**OM [17526703]**	?	C	P	P	P	P
bamB	☑	☑	☑	☑	**OM [15851030]**	?	?	E	P	P	OM
bamC	☑	☑	☑	☑	**OM [15851030]**	OM	P	P	P	P	?
bamD	☑	☑	☑	☐	**OM [15851030]**	?	E	P	P	P	P
blc	☑	☑	☑	☐	**OM [7559452]**	?	OM	E	P	C	P
borD	☑	☑	☑	☐	**M ***	?	?	P	C	C	P
csgG	☑	☑	☑	☑	**OM [19011034]**	?	?	P	P	C	OM
cusC	☑	☑	☑	☑	**OM [11566977]**	OM	OM	E	E	P	OM
ecnA	☑	☑	☑	☐	**M [9677290]**	P	IM	E	C	?	?
ecnB	☑	☑	☑	☐	**M [9677290]**	P	E	E	C	?	?
emtA	☑	☑	☑	☐	**M [9642199]**	E	P	P	P	P	?
flgH	☑	☑	☑	☑	**OM [12500982]**	?	OM	E	E	P	OM
gfcB	☑	☑	☑	☐	**OM [16030220]**	?	P	P	C	P	OM
gfcE	☑	☑	☑	☐	**OM [16030220]**	OM	OM	P	C	P	?
lolB	☑	☑	☑	☐	**OM [9384574]**	?	OM	P	P	P	OM
lpp	☑	☑	☑	☑	**OM [4261992]**	?	E	E	P	P	P
mdtE	☑	☑	☑	☑	**M [11566977]**	M	IM	P	C	P	OM
metQ	☑	☑	☑	☐	**P [12819857]**	?	P	P	P	P	P
mltA	☑	☑	☑	☐	**OM [9287002]**	?	E	P	E	E	P
mltB	☑	☑	☑	☐	**OM [9287002]**	M	E	P	P	P	E
mltC	☑	☑	☑	☐	**OM [9158737]**	E	E	C	E	OM	?
mltD	☑	☑	☑	☑	**M [10843862]**	?	E	P	P	P	OM
nlpA	☑	☑	☑	☐	**IM [2841301]**	?	IM	P	C	C	P
nlpC	☑	☑	☑	☐	**M [9573210]**	?	?	P	E	C	P
nlpD	☑	☑	☑	☑	**OM [15513925]**	?	E	P	P	P	?
nlpI	☑	☑	☑	☐	?	?	E	E	C	C	P
osmB	☑	☑	☑	☐	?	?	P	E	E	E	E

osmE	☑	☑	☑	☐	?	?	?	P	P	P	P
pal	☑	☑	☑	☐	**OM [11115123]**	OM	OM	P	P	E	OM
rcsF	☑	☑	☑	☐	**OM [13129944]**	?	?	P	P	P	P
rlpA	☑	☑	☑	☑	**IM ***	?	P	P	P	P	P
slp	☑	☑	☑	☐	**OM [8022277]**	OM	P	P	P	P	?
slyB	☑	☑	☑	☐	**OM ***	OM	E	E	E	P	?
spr	☑	☑	☑	☐	?	?	?	E	E	P	OM
vacJ	☑	☑	☑	☐	**OM [19383799]**	?	OM	E	C	P	P
wza	☑	☑	☑	☐	**OM [15090537]**	OM	IM	P	P	P	OM
yafT	☑	☑	☑	☐	?	?	P	P	P	P	P
yafY	☑	☑	☑	☐	**IM [15252048]**	?	P	P	C	OM	?
ybaY	☑	☑	☑	☐	?	P	P	P	P	P	?
ybfN	☑	☑	☑	☐	?	?	?	P	P	E	P
ybfP	☑	☑	☑	☐	?	?	P	P	P	P	E
ybhC	☑	☑	☑	☑	**OM [10806384]**	?	E	E	P	P	OM
ybjP	☑	☑	☑	☐	?	?	?	E	P	P	P
ycaL	☑	☑	☑	☐	?	E	E	E	E	OM	?
yceB	☑	☑	☑	☐	?	?	?	P	P	C	?
yceK	☑	☑	☑	☐	?	?	?	IM	C	C	C
ycfM	☑	☑	☑	☐	?	?	E	P	P	P	P
ycjN	☑	☑	☑	☐	**P ***	P	P	P	P	P	P
ydcL	☑	☑	☑	☐	?	?	P	P	P	P	P
yeaY	☑	☑	☑	☐	?	?	OM	P	P	P	E
yecR	☑	☑	☑	☐	?	?	?	E	E	C	E
yedD	☑	☑	☑	☐	?	?	P	P	P	P	?
yehR	☑	☑	☑	☐	?	?	E	P	P	OM	?
yfeY	☑	☑	☑	☐	?	?	?	P	P	P	OM
yfgH	☑	☑	☑	☐	?	?	OM	E	E	E	OM
yfiB	☑	☑	☑	☐	?	OM	OM	P	E	P	OM
yfiL	☑	☑	☑	☐	?	?	P	E	P	E	?
yfjS	☑	☑	☑	☐	**IM [15252048]**	?	P	P	C	OM	?
ygdI	☑	☑	☑	☐		?	E	P	P	P	?

ygdR	☑	☑	☑	☐	?	?	?	P	C	C	OM
ygeR	☑	☑	☑	☐	?	E	E	E	E	OM	?
yghG	☑	☑	☑	☐	?	?	P	P	E	E	E
yhdV	☑	☑	☑	☐	?	?	IM	P	P	P	P
yhfL	☑	☑	☑	☐	?	?	?	E	E	C	?
yiaD	☑	☑	☑	☑	?	OM	E	E	E	OM	?
yiiG	☑	☑	☑	☐	?	?	?	P	P	P	P
ynbE	☑	☑	☑	☐	?	?	?	C	C	C	C
ynfC	☑	☑	☑	☐	?	?	P	P	C	E	?
yoaF	☑	☑	☑	☐	?	?	?	P	P	P	OM
yqhH	☑	☑	☑	☐	?	?	OM	P	C	C	P
apbE	☑	☐	☑	☐	**P [10572132]**	?	P	P	P	P	?
hslJ	☑	☐	☑	☐	?	?	?	P	P	P	P
nlpE	☑	☐	☑	☐	**OM [7635808]**	?	OM	P	P	P	P
yddW	☑	☐	☑	☐	?	?	?	P	P	P	P
yjbF	☑	☐	☑	☐	?	P	P	P	P	P	?
yraP	☑	☐	☑	☑	**P**	P	P	P	P	C	P
rlpB (lptE)	☑	☐	☑	☐	**OM [16861298]**	?	?	P	P	P	OM
yjeI	☑	☐	☑	☐	?	?	E	E	E	P	?
pgaB	☑	☑	☐	☐	**M ***	?	?	P	C	C	P
cyoA	☐	☑	☑	☐	**IM [16481320]**	M	IM	P	C	P	IM

Table 1B					*** Ecocyc information**
	**[LVI]**	**[ASTVI]**	**[ASG]**	**C**	
yjeI	*M*	A	G	C	***Inner Membrane***
apbE	*F*	*V*	G	C	***Outer Membrane***
hslJ	*M*	A	G	C	***Membrane***
nlpE	L	*M*	G	C	
yddW	L	*L*	*L*	C	***Undetermined***
yjbF	L	*Q*	A	C	***Extracellular***
yraP	L	*Q*	G	C	***Cytoplasmic***
rlpB (lptE)	*T*	A	G	C	***Periplasmic***
